# Shaping the glioblastoma microenvironment to enhance CAR-NK immunotherapy

**DOI:** 10.3389/fimmu.2025.1681966

**Published:** 2025-12-01

**Authors:** Emily Tang, Jake Y. Chen

**Affiliations:** 1 North Carolina School of Science and Mathematics, Durham, NC, United States; 2 Systems Pharmacology Artificial intelligence (AI) Research Center and Department of Biomedical Informatics and Data Science, School of Medicine, The University of Alabama at Birmingham, Birmingham, AL, United States

**Keywords:** glioblastomas, microenvironment, chimeric antigen receptor, natural killer cell, immunotherapy

## Abstract

Chimeric antigen receptor–natural killer (CAR-NK) cell therapy has shown favorable results in treating hematological malignancies but with limited efficacy against solid tumors, including glioblastomas, which is partly due to the immunosuppressive microenvironment of solid tumors. This mini review focuses on the various immunosuppressive strategies employed by the glioblastoma microenvironment for immune evasion, including stromal barriers, hypoxic conditions, immunosuppressive cytokines, downregulation of activating ligands, and upregulation of immune checkpoints. A range of emerging strategies has been proposed to counteract these inhibitory effects, such as genetic engineering of NK cells and molecular targeting of the stroma in combination with oncolytic virus therapy. Future single-cell spatiotemporal omics studies are expected to further enable a personalized and dynamic approach to treating glioblastoma with improved outcomes.

## 1 Introduction

Glioblastomas are among the most aggressive and treatment-resistant brain tumors despite advancements in surgery, chemotherapy, and radiation therapy. One promising immunotherapy for glioblastomas is chimeric antigen receptor T cell (CAR-T) therapy to target tumor cells with the immune cell’s innate cytotoxic activity. However, CAR-T therapy has shown limited success and a high rate of recurrence in clinical studies (1). The emergence of chimeric antigen receptor natural killer (CAR-NK) therapy provides a way to counteract the limitations of previous treatments. Compared with CAR-T, CAR-NK therapy has off-the-shelf potential and better safety, such as reduced risk of cytokine release syndrome (CRS) and graft versus host disease (GvHD) (2, 3). However, the efficacy of CAR-NK therapy is compromised by the tumor microenvironment (TME), which limits its application in various tumors (2). The goal of this article is to explore how the glioblastoma TME affects natural killer cells and ways of targeting the TME to enhance CAR-NK therapy in glioblastomas.

## 2 Characteristics of glioblastomas and its immunosuppressive microenvironment

Glioblastomas are characterized by their immunosuppressive microenvironment, which contains endothelial cells, astrocytes, immune effector cells like microglia, myeloid-derived suppressor cells (MDSCs), tumor-associated macrophages (TAMs), regulatory T-cells (Tregs) and other noncellular components (4, 5). This environment is a complex interplay of tumor, immune, and molecular features. The cellular components are known to contribute to the suppressive environment by fostering tumor evasion, progression, and angiogenesis (4). For example, MDSCs are known to promote tumor growth by secreting certain factors, such as tumor necrosis factor (TNF)-α and vascular endothelial growth factor (VEGF) (6). Immunosuppression may be exacerbated by hypoxia, cytokines (IL4, IL10, and TGF-β), and varying expression of MHC class I molecules (5, 7), which aids in impairment of recognition by immune cells and immune invasion (7). The blood-brain barrier (BBB) is another anti-immune mechanism utilized by glioblastomas. The BBB is a membrane composed of microvascular endothelial cells that separate blood from brain interstitial fluid. For treatment to be administered, the BBB must be penetrated, posing a challenge to many therapies (8). The BBB is among one of a glioblastoma’s characteristics that presents a hurdle to treatment, and it is known that the BBB may be disrupted in certain pathological states that allow immune traffic to more easily infiltrate the central nervous system (9). Clinically, intratumoral/resection cavity and intracerebroventricular (ICV) administration, as well as focused ultrasound and convection-enhanced delivery, have been explored for treating glioblastoma with varying results (10, 11). Overall, the glioblastoma microenvironment contributes to reduced treatment efficacy through various immunosuppressive/evasive mechanisms and the BBB as a physical barrier. This tumor microenvironment represents a potential target for further modulation to enhance antitumor therapy.

## 3 Biology and function of NK cells in the central nervous system

Natural killer (NK) cells make up a small subset of immune cells in the brain and glioblastomas, consisting of about 1.5% of the total immune cells in a healthy CNS. In the healthy brain, NK cells perform a variety of immune functions: they participate in immune surveillance, work as cytotoxic effectors, and regulate inflammation (12). scRNA-seq analysis has revealed five NK subtypes: CD56bright, early CD56dim, intermediate CD56dim, late CD56dim and adaptive, all named to reflect their maturation. The two different cell states, CD56 bright and dim are notably distinct due to their differential expression of the CD56 neural cell adhesion molecule gene (NCAM1) (13). CD56 bright NK cells are considered precursors to the more mature CD56 dim NK cells. Current studies support that bright cells play a bigger cytokine effector role while dim cells are known for their cytotoxic ability (14, 15). The infiltration of NK cells into the central nervous system is poorly investigated, but research shows that higher NK migration occurs during BBB breakdown (12). In cerebral small vessel disease (CSVD), proteomic analyses revealed that CD56 dim NK cells may contribute to BBB breakdown through secretion of cathepsin D (CTSD), a molecule that participates in protein degradation. This allows the NK cells to further infiltrate and disrupt the neural environment, suggesting that NK cells may intensify damage in cerebrovascular diseases (16, 17). Indeed, NK cell trafficking is very complex and is influenced by many different factors, such as adhesion and cytokine networks secreted by NK, glial, vascular, and other CNS cells (12, 18, 19).

## 4 CAR-NK therapy: current landscape and glioblastomas-specific challenges

### 4.1 CAR-NK therapy and its cellular sources

The CAR construct is composed of a few domains: an extracellular antigen-binding domain, transmembrane domains, intracellular signaling domains, and hinge regions (20). Majority of NK cells used in therapy are sourced from the NK92 cell line due to its “off the shelf” potential, lower manufacturing cost, and reduced sensitivity to freeze/thaw cycles. However, the drawbacks of this line include its tumorigenic potential and loss of expansion due to lethal irradiation prior to infusion (21, 22). Other prospective cell lines include peripheral blood mononuclear cells (PBMCs), which have been used in many clinical studies; umbilical cord blood (UCBs) due to the advantage of selecting donors with HLA compatibility and desired NK receptor traits; CD34+ hematopoietic progenitor cells (HPCs), which can be obtained in large amounts and exhibit high cytotoxicity in certain cancers such as leukemias; and induced pluripotent stem cells (iPSCs) for creating “off the shelf products (23)”.

### 4.2 CAR-NK immunotherapy success in hematological malignancies

The focus for treatment in glioblastomas has shifted to CAR-NK therapy due to successes in hematological malignancies. Several clinical trials (ClinicalTrials.gov) have been conducted to evaluate the efficacy of CAR-NK therapy in hematological tumors, such as leukemias, and has shown clinical significance (20). For instance, a study of CNTY-101 (an iPSC-derived anti-CD19 CAR-NK cell product) in CD19-Positive B-Cell malignancies demonstrated initial safety and efficacy (NCT05336409). Another first-in-human clinical trial of CD19-CAR-UCB-NK for relapsed or refractory CD19-positive non-Hodgkin’s lymphoma or chronic lymphocytic leukemia (CLL) was conducted in 11 patients. The results were mostly positive, with eight out of 11 patients responding to the treatment: seven achieved complete remission, and one had a partial remission. (NCT03056339).

### 4.3 CAR-NK challenges in solid tumors

While CAR-NK therapy provides many benefits, evident in hematological malignancies, its effectiveness is limited by certain barriers in solid tumors. **Table 1** lists some recently completed as well as ongoing clinical trials on CAR-NK therapy for hematological malignancies and solid tumors. However, no outcomes have been reported for glioblastoma treatment yet. Several mechanisms are known to contribute to the reduced effect in solid tumors: hypoxia and other metabolite factors, NK-tumor interactions, cytokines, and exosomes (2).

**Table 1:** Clinical trials treating hematological malignancies and solid tumors with CAR-NK therapy.

NCT Number	Title	Disease	Phase	Outcome
NCT05336409	A Study of CNTY-101 in Participants With CD19-Positive B-Cell Malignancies (ELiPSE-1)	B-Cell Malignancies	Phase 1	ORR/CRR was 67%/33% for 300e6 cells (DL2A)
NCT03056339	Umbilical & Cord Blood (CB) Derived CAR-Engineered NK Cells for B Lymphoid Malignancies	Relapsed or refractory CD19-positive non-Hodgkin’s lymphoma or chronic lymphocytic leukemia	Phase 1/2	8/11 responded to treatment, 7 achieved complete remission
NCT05472558	Clinical Study of Cord Blood-derived CAR-NK Cells Targeting CD19 in the Treatment of Refractory/Relapsed B-cell NHL	Relapsed/Refractory B-Cell Non-Hodgkin Lymphoma	Phase 1	62.5% response rate at day 30, 4/8 patients achieved a complete response
NCT06696846	CD70-CAR-NK Cell Therapy for T Cell Lymphoma and Acute Myeloid Leukemia	T-Cell Lymphoma, Acute Myeloid Leukemia	Phase 1	Active
NCT06690827	Clinical Trial of CD123-targeted CAR-NK Therapy for Relapse/refractory AML or BPDCN	Acute Myeloid Leukemia, Blastic Plasmacytoid Dendritic Cell Neoplasm	Phase 1	Active
NCT06572956	Clinical Study on the Safety and Efficacy of CAR-T/CAR-NK Cells in the Treatment of Recurrent Refractory or Unresectable Solid Tumors	Solid tumors, including pancreatic, prostate, breast, glioma, etc.	Early Phase 1	Active
NCT06454890	Clinical Study of Trop2 CAR-NK in the Treatment of Relapsed/Refractory Non-Small Cell Lung Cancer (NSCLC)	Non-Small Cell Lung Cancer	Phase 1/2	Active
NCT05410717	CLDN6/GPC3/Mesothelin/AXL-CAR-NK Cell Therapy for Advanced Solid Tumors	AXL-Positive Advanced Solid Tumors including ovarian, testis, endometrial, etc.	Phase 1	Active
NCT06856278	Clinical Study of NKG2D CAR-NK Combined with PD-1 Monoclonal Antibody in the Treatment of ATC	Anaplastic Thyroid Carcinoma	Phase 1/2	Active

ORR, overall response rate; CRR, complete response rate.

Hypoxia is the process involving abnormal changes in metabolism due to insufficient oxygen supply. In the TME, hypoxia reduces ERK and STAT3 phosphorylation through protein tyrosine phosphatase 1 (SHP-1) dependent manner, diminishing NK cell cytotoxicity by up to 40% as measured by flow cytometry in an *in vitro* system (24). In addition, hypoxia promotes angiogenesis, metabolic reprogramming, immune evasion, inflammation, and genomic instability (25).

NK-tumor cell interaction also plays a role in reducing the efficacy of CAR-NK therapy in solid tumors. NKG2D is a surface receptor of NK cells and can stimulate immune effectors without antigen presentation (26). As a result, NKG2D plays a crucial role in immune activation; however, it may be downregulated due to the production of soluble NKG2DLs by tumor cells, therefore preventing NK-tumor cell association and diminishing the efficacy of CAR-NK therapy (2, 27).

Cytokines are signaling proteins that mediate communication within the immune system. Transforming growth factor-β (TGF-β) regulates cell growth and differentiation. It has been found that TGF-β is mainly produced by tumor cells and is related to the poor prognosis in solid tumors like lung, gastric, liver, and pancreatic cancer (28–30). TGF-β suppresses NK cell activation and function by downregulating activating receptors and inhibiting the mTOR pathway (31).

Exosomes, vesicles that contain various lipids and proteins, mediate communication and play an important role in disease prognosis (32). Tumor derived exosomes (TDEs) differ from normal exosomes in their biological function: they are involved in tumor formation, metastasis, angiogenesis, and are known to reprogram immune cells via inhibitory proteins, cytokines, RNA, and other factors, such as in non-small cell lung cancer, leukemia, and hepatocellular carcinoma (33–36). Glioblastoma releases exosomes containing 4IgB7H3, which can suppress NK-mediated tumor lysis (37). TDEs also negatively affect CAR-NK therapy by dysregulating NK cell function and reducing their antitumor activity (38).

## 5 How the glioblastomas TME impairs NK function

### 5.1 Suppression by astrocytes and cancer associated fibroblasts

Glioblastomas are known for their suppressive TME and BBB that impedes immune infiltration. Astrocytes and microglia have been shown to alter cellular arrangement and contribute to a physical barrier in 3D models. Spheroid models were used to model the tumor, and pure spheroid models had loosely packed cells that exhibited large pores on the surface. The introduction of astrocytes and microglia caused the model to become more compact and structured in certain areas. The nuclear density was higher in these models and supports that these cells may alter the glioblastoma morphology to reduce infiltration (39). In addition, cancer associated fibroblasts (CAFs) are known to exhibit immunosuppressive properties and promote tumor progression. CAFs are a common characteristic of the TME and may impair the cytotoxic function of NK cells (40). This occurs through ferroptosis, a cell death process caused by build-up of iron-catalyzed lipid peroxides (41). A study in gastric cancer found that CAFs increase the intracellular reactive oxygen species (ROS) and malondialdehyde (MDA) lipids within NK cells, which has been shown to consistently contribute to CAF-induced death in NK92 cells. As expected, cell death was reversed by ferroptosis inhibitor Ferrostatin-1 (Fer-1) and cytotoxicity was partially repaired by Fer-1 and Lip-1, another ferroptosis inhibitor (42). In glioblastoma, enhanced ferroptosis was shown to attenuated antitumor cytotoxic killing of immune cells (43). Lastly, it should be noted that CAFs in glioblastoma may differ from those in epithelial tumors, as the fibroblast-like stromal cells or perivascular fibroblasts in glioblastoma may act as potential glioblastoma-initiating cells within the TME (44).

### 5.2 Impairment by indoleamine 2,3-dioxygenase, TGF-β, and adenosinergic processes

Indoleamine 2,3-dioxygenase (IDO) is an enzyme that facilitates the metabolism of tryptophan (Trp) and is controlled in the glioblastoma microenvironment to suppress immune activity. IDO-1 in the glioblastoma TME reduces tryptophan levels, which has an immunosuppressive effect and leads to CD8+ T cell exhaustion (45). In thyroid cancer, cancer cells produce kynurenine using IDO, resulting in NK dysfunction. Kynurenine enters the NK cell via the aryl hydrocarbon surface receptor and disrupts NK receptor expression through modulation of the STAT1 and STAT3 pathways (46). A recent study using the kynurenine inhibitor PVZB3001 demonstrated promising results, as this compound restored NK cell viability and function in A172 glioblastoma cell cultures (47). In addition to IDO/kynurenine, TGF-β and activin A inhibit NK cell function by reducing its efficacy in tissue homing and residency. Also, CD73-mediated production of adenosine drives reprogramming of the TME and contributes to immune evasion in solid tumors. Increased activity of CD39 and CD73 drives the adenosinergic process by converting ATP to AMP and then to adenosine, which suppresses immune responses (48). NK cells can be engineered to target the CD73-adenosine axis and block the immunosuppressive effect (49).

### 5.3 Downregulation of activating ligands

The NKG2D ligand family acts as activating receptors for immune responses in NK cells and some T cell. It has been a target in potentially improving cancer immunotherapy because of its selective expression and strong NK cell activating potency (50). However, glioblastomas stem cells (GSCs) in glioblastomas downregulate NKG2D ligands, which impairs NK cell-driven killing (51).

### 5.4 Upregulation of immune checkpoints

PD-L1 is an immunosuppressive receptor and is mainly located on immune cells such as macrophages, CD3+/CD8+ T cells, and NK cells. The expression of PD-1/PD-L1 in glioblastomas is upregulated and often contribute to immune escape. PD-L1 expression is elevated at the edge of glioblastomas tumor cells compared to the tumor core, suggesting the presence of a PD-L1-mediated barrier at the tumor margins that impedes immune cell infiltration (52). This is another line of evidence that the glioblastomas TME plays a significant role in impairing immune cell function.

## 6 Opportunities: reprogramming the TME to enhance CAR-NK therapy

### 6.1 Engineering CAR-NK cells to resist TGF-β and hypoxia

Recently, there have been many strategies proposed to address the limitations of CAR-NK immunotherapy. Mentioned previously, TGF-β impairs the antitumor activity of NK cells; now, studies show that NK cells engineered with the CRISPR-Cas9 system to knock SMAD4 exhibit increased resistance to TGF-β suppression (53, 54). The canonical TGF-β signaling pathway in NK cells is mediated by SMAD2 and SMAD3 phosphorylation, subsequently followed by the phosphorylated SMAD2/3 forming a complex with SMAD4. SMAD4 plays a central role in TGF-β signaling and enables the SMAD2/3 complex to influence gene expression in the nucleus. The knockout of the SMAD4 gene prevents the SMAD2/3 complex from forming a functional transcriptional complex and blocks the signaling pathway to the nucleus. Experimental data shows that NK cells with reduced or absent SMAD4 are less susceptible to TGF-β-induced downregulation of activating receptors and retain higher cytotoxic function (54–56).

Hypoxia is another hurdle in CAR-NK efficacy. Studies found that HER1-overexpressing NK cells genetically engineered with catalase, called HER1-CAR-CAT-NK cells, were more tolerant towards hypoxia and high levels of oxidative stress. Intratumoral delivery of HER1-CAR-CAT-NK cells resulted in sustained attenuation of tumor hypoxia and improved retention and antitumor activity of the engineered NK cells (57). Other strategies for overcoming the hypoxic TME include direct oxygen delivery to tumors, increasing intratumoral oxygen levels, and combinatorial approaches targeting pH, angiogenesis, and immune dysfunction (58).

### 6.2 Combination treatments, genetic engineering, and oncolytic viruses

Other advancements have come out by genetic manipulation and co-administering drugs to ameliorate the functional defects of NK cells in the TME. Since activation of the CXCL12/CXCR4 axis restricts autophagy, an essential cellular process in NK cells, inhibition of CXCR4 and C/EBPβ restores NK functionality (59). This can be seen as a strategy that can be employed when developing NK products for CAR-NK therapy to improve efficacy and resistance to the TME.

Hypoxic tumors overexpress proteins like VEGF, which influence angiogenesis and promotes immune escape. Combination therapy with anti-angiogenic drugs targeting VEGF may be considered to normalize vascular structure and improve the efficacy of CAR-NK therapy. However, it is known that monotherapy with anti-angiogenic drugs can exacerbate the immunosuppressive TME and result in poor prognosis (2). Recently, studies have found that administering oncolytic viruses engineered to secrete immunostimulatory cytokines improves the efficacy of CAR-NK therapy in neuroblastoma and other cancers. The modified herpes simplex virus C021 expresses interleukin-21 (IL-21), which boosts NK cell proliferation and persistence. In addition, the combination of C021 with anti-ROR1 CAR-NK cells significantly increased cytotoxicity against neuroblastoma cells (60). The C021 virus effectively reprograms the TME from a cold (immunosuppressive) to hot (immunostimulatory) environment and enhances immune infiltration. As a result, the integration of cytokine-secreting oncolytic viruses with CAR-NK cells provides a synergistic and novel immunotherapeutic approach for enhancing anti-tumor efficacy (61).

### 6.3 Targeting CAFs and HER2 expressing cells

Some recent developments have turned attention to stromal players that contribute to immunosuppression in the TME. The presence of CAFs in the TME are associated with poor prognosis and research has shown that combination therapy with CAF inhibitors enhanced CAR-NK cytotoxicity (62). CAR-NK-cP6 cells modified to continuously produce the P6 peptide disrupt the interaction between TGF-β1 and its receptor TGF-βR1. CAFs are known to secrete TGF-β1, a major immunosuppressive cytokine which creates a physical and molecular barrier for immune surveillance (63). The modified cells block TGF-β1 signaling in CAFs through a paracrine effect, therefore allowing CAR-NK-cP6 cells to display cytotoxic efficacy in a CAF-rich environment. Targeting TGF-β can provide synergistic effects for eliminating CAFs, cancer cells, and their immunosuppressive effect on immune cells (64).

HER2 is expressed in many tumors, such as glioblastomas and breast cancers. Moderate to high HER2 expression has been detected in 41% of primary glioblastoma samples and in the majority of investigated glioblastoma cell lines, as shown by immunohistochemistry (65). As a result, HER2 has been a target for CAR-NK therapy in glioblastomas; however, monotherapy is not enough to overcome the immunosuppressive TME in advanced-stage glioblastomas. Combination therapy of NK-92/5.28.z CAR-NK cells and anti-PD-1 checkpoint inhibition demonstrated robust anti-tumor activity and was able to selectively lyse HER2-expressing glioblastomas cells *in vitro*. Checkpoint blockade PD-1 has a considerable effect due its ability to block the pathways of immune response. Recent studies have identified PD-1^+^ NK cell subsets with impaired immune functions in tumors such as high-grade serous ovarian cancer and B-cell chronic lymphocytic leukemia (B-CLL), making them suitable targets for combined immune checkpoint blockade therapy. Administration of PD-1 inhibitors allows both CAR-NK cells and endogenous T cells to proliferate and maintain their cytotoxic functions (66–68). In mouse models, the modified CAR-NK cells were capable of eradicating smaller HER2 tumors and developed durable immunity against glioblastomas rechallenge (69).

## 7 Discussion

CAR-NK immunotherapy relies on the innate cytotoxic and targeting ability of NK cells in tumors. The key challenges in glioblastomas include the stromal barriers, hypoxia environment, immune-inhibitory cytokines, downregulation of activating ligands, and upregulation of immune checkpoints. Recent advancements have begun addressing these limitations with promising outcomes, such as modifying NK cells to resist hypoxic environments, modulating stromal elements, and combination treatments.

Future work is needed to further improve the field of CAR-NK therapy. Given the heterogeneity of the glioblastomas TME, patient-specific profiling using single-cell and spatial tools will be crucial for identifying immune escape mechanisms and elucidating novel combination strategies. These high-resolution tools can reveal spatial architecture of stromal cells, ligand-receptor interactions, and immune infiltrates, allowing for clear-cut design of CAR constructs tailored for TME landscapes. More specifically, upcoming efforts could include: (i) pairing spatial ligand–receptor maps to select CAR antigens and cytokine payloads; and (ii) using patient-matched organoid or spheroid co-cultures with astrocytes and microglia to benchmark NK cell persistence and motility under hypoxia. Overall, integrating CAR-NK therapy with TME reprogramming will be essential for overcoming immunosuppression and enhancing infiltration, persistence, and cytotoxicity of NK cells. With ongoing biological and therapeutic progress, CAR-NK therapy will transition from a generalized strategy to a personalized and dynamic approach for treating glioblastomas.
